# Editorial: Unraveling immune metabolism: single-cell & spatial transcriptomics illuminate disease dynamics

**DOI:** 10.3389/fendo.2026.1779505

**Published:** 2026-01-23

**Authors:** Yejun Tan, Yafeng Zhu, Zhengtao Liu

**Affiliations:** 1 Department of Health Technology and Informatics, The Hong Kong Polytechnic University, Hong Kong, Hong Kong SAR, China; 2 Guangdong Provincial Key Laboratory of Malignant Tumor Epigenetics and Gene Regulation, Guangdong-Hong Kong Joint Laboratory for RNA Medicine, Medical Research Center, Sun Yat-Sen Memorial Hospital, Sun Yat-Sen University, Guangzhou, China; 3 Key Laboratory of Artificial Organs and Computational Medicine in Zhejiang Province, Shulan International Medical College, Zhejiang Shuren University, Hangzhou, China; 4 NHC Key Laboratory of Combined Multi-Organ Transplantation, Key Laboratory of the Diagnosis and Treatment of Organ Transplantation, First Affiliated Hospital, School of Medicine, Zhejiang University, Hangzhou, China

**Keywords:** immune microenvironment, immunometabolism, metabolic reprogramming, scRNA sequencing, spatial transcriptomics

## Introduction

The interplay between cellular metabolism and immune function—immunometabolism—has emerged as a cornerstone of modern pathology (1). Immune cells are not static entities; they continuously adapt their metabolic programs to survive and function within hostile microenvironments, whether in the hypoxic core of a tumor, the inflamed synovium of an arthritic joint, or the fibrotic tissue of a failing kidney (1–5). Historically, our understanding of these processes was limited by bulk analyses that averaged metabolic signals across heterogeneous cell populations (6). However, the advent of single-cell RNA sequencing (scRNA-seq) and spatial transcriptomics has precipitated a paradigm shift (6, 7). We can now dissect the metabolic heterogeneity of immune cells at high resolution, mapping how specific metabolic pathways drive disease progression, resistance to therapy, and tissue remodeling (6).

This Research Topic, Unraveling Immune Metabolism: Single-Cell & Spatial Transcriptomics Illuminate Disease Dynamics, was curated to bridge the gap between static metabolic profiling and dynamic disease pathology. The Research Topic published here spans a diverse spectrum of conditions—from solid tumors and renal disease to autoimmune disorders and cardiovascular failure. Collectively, they demonstrate how metabolic rewiring is not merely a consequence of disease, but a fundamental driver of the immune landscape.

## Reshaping the tumor microenvironment

Nowhere is metabolic competition more fierce than in the tumor microenvironment (TME), where cancer cells and immune cells vie for limited nutrients. Several contributions to this topic highlight how spatial and single-cell technologies are decoding this competition.

In the context of colorectal cancer, Wang et al. utilized single-cell and spatial transcriptomics to construct a high-resolution map of tumor heterogeneity. Their work reveals distinct molecular programs that govern the spatial distribution of immune cells, offering new targets for disrupting the tumor-supportive niche. Similarly, Fu et al. investigated lung adenocarcinoma, identifying the Midkine (MDK)-Nucleolin (NCL) pathway as a critical regulator of the immunosuppressive environment. By integrating spatial data, they demonstrated how this pathway orchestrates immune exclusion, suggesting that metabolic or signaling interventions targeting MDK-NCL could reinvigorate anti-tumor immunity.

Two comprehensive reviews further elucidate the metabolic hurdles within the TME. Chen et al. focused on gastric cancer, detailing how aberrant lipid metabolism reshapes the immune microenvironment to favor tumor growth. Chen et al. extended this discussion to Triple-Negative Breast Cancer (TNBC), synthesizing evidence on how metabolic plasticity limits the efficacy of immunotherapy and proposing metabolic vulnerabilities that could be exploited for combined treatment strategy.

## Metabolic reprogramming in renal and systemic disease

Beyond oncology, this topic emphasizes the critical role of immunometabolism in chronic inflammatory and metabolic diseases. The progression from Acute Kidney Injury (AKI) to Chronic Kidney Disease (CKD) represents a complex metabolic shift. Zeng et al. applied integrated transcriptomics to identify key genes—CLCNKB, KLK1, and PLEKHA4—that mark this transition, providing potential biomarkers for early intervention. Complementing this, Li et al. employed a multi-omics and network pharmacology approach to validate the Jianpi-Yishen formula, a traditional intervention, revealing its capacity to modulate metabolic networks in CKD.

In the realm of systemic metabolic disorders, Li et al. utilized scRNA-seq to explore Type 2 Diabetes Mellitus (T2DM). Their study uncovers distinct immunometabolic alterations in peripheral blood mononuclear cells, linking specific immune subtypes to the systemic metabolic dysregulation characteristic of diabetes.

## Autoimmunity, inflammation, and stress responses

The plasticity of macrophages and T cells is central to autoimmune pathology. Jiang et al. provided a compelling analysis of Rheumatoid Arthritis (RA), specifically the ACPA-negative subtype. Their scRNA-seq analysis highlighted a unique macrophage expansion driven by metabolic reprogramming, distinguishing the pathogenesis of this subtype from classical RA and suggesting that metabolic inhibition could be a viable therapeutic avenue for these patients.

Finally, the Research Topic addresses how immune metabolism responds to systemic stress and hypoxia. Wang et al. probed heart failure through the lens of immunogenic cell death (ICD), identifying transcriptomic biomarkers that link cell death pathways to immune activation in cardiac tissue. In a study connecting hypoxia to systemic inflammation, Ye et al. used interpretable machine learning to decode the “hypoxia-exosome-immune triad” in Obstructive Sleep Apnea (OSA). They revealed how the PRCP/UCHL1/BTG2 axis drives metabolic dysregulation, offering a novel mechanistic view of how sleep-disordered breathing impacts immune health.

## Conclusion

The studies presented in Unraveling Immune Metabolism collectively reinforce the concept that metabolism is not merely the energy source for immune cells, but the instruction manual for their function. By leveraging single-cell and spatial technologies, these authors have moved beyond static snapshots to reveal the dynamic, location-specific metabolic engines driving disease. As we look to the future, the integration of these transcriptomic maps with direct metabolite sensing and flux analysis will be the next frontier, promising precision therapies that target the metabolic heartbeat of pathology.
